# Tricuspid Aortic Valve Reconstruction with Autologous Pericardium (Ozaki Technique) in Bicuspid Aortic Valve Infective Endocarditis

**DOI:** 10.21470/1678-9741-2021-0630

**Published:** 2023

**Authors:** Antonio D’Errico Ramirez, Concetta Losito, Nicola Di Bari, Lorenzo Giovannico, Tommaso Acquaviva, Roberta Romito, Aldo Domenico Milano

**Affiliations:** 1 Division of Cardiac Surgery, Department of Emergency and Organ Transplant, Policlinico Hospital, University of Bari, Bari, Italy; 2 Division of Cardiology, Policlinico Hospital, University of Bari, Bari, Italy

## CASE PRESENTATION

A 25-year-old Gambian man was admitted for persistent hyperpyrexia, worsening dyspnea and asthenia, and new-onset cardiac murmur at routine control. He had no past medical history.

**Fig. 1:**
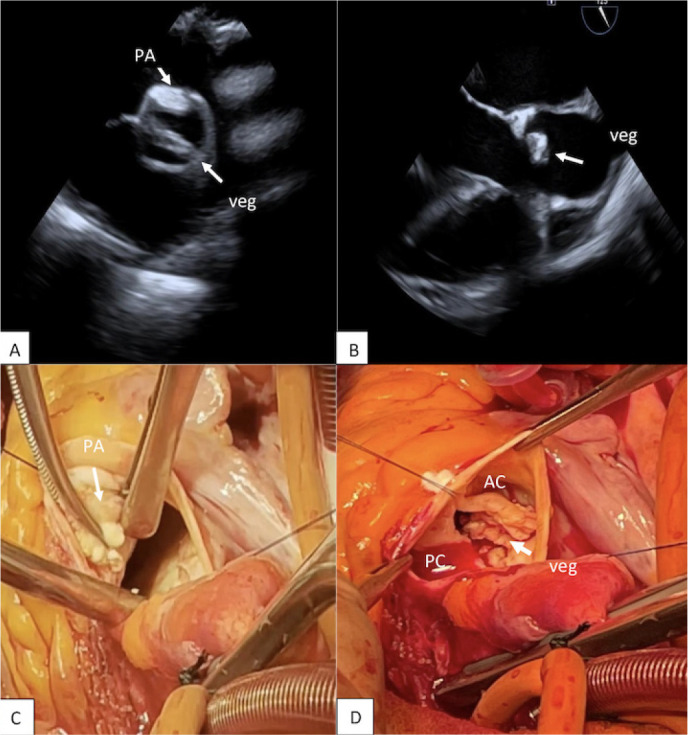
A) Preoperative transesophageal echocardiography (TTE), short axis (aortic periannular abscess [PA], anterior leaflet vegetation [veg]). B) Intraoperative TTE (bicuspid aortic valve, mobile veg on anterior leaflet). C) Intraoperative view: PA under the anterior commissure of bicuspid aortic valve. D) Intraoperative view: bicuspid aortic valve (Sievers type 0 anterior-posterior) with a large mobile vegetation on the ventricular side of the anterior cusp (AC). PC=posterior cusp.

At the admission, transesophageal echocardiography (TEE) demonstrated a left ventricular ejection fraction of 60% with a Sievers type 0 anterior-posterior (A-P) bicuspid aortic valve (BAV) with severe regurgitation (vena contracta [VC]: 7 mm; pressure half-time: 92 msec; effective regurgitant orifice area: 35 mm2; right ventricle: 70 ml), a 13-mm mobile vegetation on the ventricular side of the anterior valve leaflet (Figure 1), a dilated left ventricle (left ventricular end-systolic dimension: 55 mm) and severe mitral regurgitation (VC: 12 mm) due to papillary muscles tethering.

Since blood cultures were negative throughout his hospital stay, empiric antibiotic treatment with ceftriaxone and vancomycin was started.

Brain computed tomography showed ischemic lesions in the frontal lobe due to septic embolism, and coronary angiography showed no abnormalities.

The patient was scheduled for urgent aortic valve neocuspidization (AVNeo) and mitral valve annuloplasty. Since aortic valve (AV) replacement with a mechanical prosthesis was excluded due to the patient’s inability to follow anticoagulant therapy as well as a homograft implantation due to unavailability, we preferred AVNeo to biological prosthesis replacement.

## TECHNICAL DESCRIPTION

**Fig. 2:**
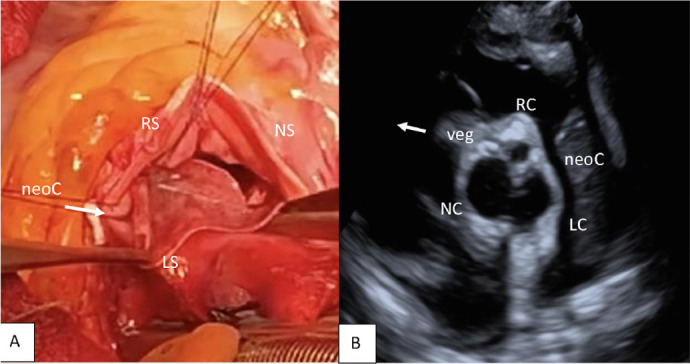
A) Intraoperative view: reconstruction of the aortic valve with autologous pericardium (Ozaki procedure); we created a new commissure (neoC) between the right and left coronary sinuses of Valsalva. B) Postoperative transesophageal echocardiography: aortic valve neocuspidization with three cusps (right cusp [RC]; left cusp [LC]; noncoronary cusp [NC]). LS=left sinus; NS=noncoronary sinus; RS=right sinus.

Operation was performed through a median sternotomy and with standard aortic/bicaval cardiopulmonary bypass. An 8×8-cm piece of pericardium was harvested, then cleaned and fixed in 0.6% glutaraldehyde solution for 10 minutes and rinsed three times in normal saline. Subsequently, we performed aortotomy and exposed the AV and confirmed the echocardiographic findings: BAV had severely retracted leaflets and a large fragile vegetation on the ventricular side of the anterior cusp. After the excision of the two leaflets, we observed a first large periannular abscess (PA) located under the anterior commissure (Figure 1) and a smaller one at the mitral-aortic junction. The abscesses were incised and drained. Then, a new commissure was designed between the two close coronary ostia, and the correct neo-commissure height was measured with a prosthesis sizer. The distances between the three new commissures, measured using the sizer designed by Ozaki, were 29 mm, 27 mm, and 25 mm, for noncoronary cusp, left coronary cusp, and right coronary cusp, respectively. New leaflets were then obtained from the pericardium and sewn to the aortic annulus using the Ozaki described procedure (Figure 2).

We also repaired the mitral valve with posterior annuloplasty using a 32-mm band of autologous pericardium.

At the end of the surgery, intraoperative TEE showed good function of the AVNeo valve and only mild regurgitation (Figure 2) and good competence of the mitral annuloplasty. (Video)

**Video 1:**
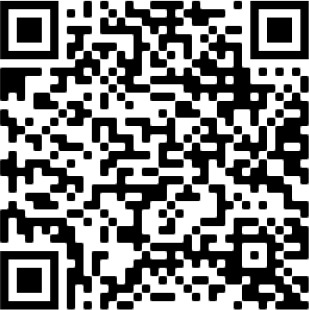
- Ozaki Technique in bicuspid aortic valve infective endocarditis surgical procedure.

The patient was discharged on the 14^th^ postoperative day with indication to follow antibiotic therapy for other four weeks and cardio aspirin for six months. No recurrence of infection occurred after one year of follow-up, and routine control echocardiogram demonstrated good function of the AVNeo valve and mitral valve.

## COMMENT

Infective endocarditis (IE) is a life-threatening condition and despite advances in antibiotic therapy, about 1/3 of patients requires a surgical treatment^[^1^]^. AV infection with PA leads to severe complications such as atrioventricular block, pseudoaneurysm, and fistula, with a perioperative mortality up to 12%^[^2^]^. Therefore, choosing the most appropriate treatment is fundamental. So far, few cases of AVNeo in IE have been described.

Using the Ozaki procedure, acceptable midterm survival and freedom from reoperation have been reported. Medium-term results have shown a reoperation rate of 4.2% and 10-year survival rate of 85.9%^[^3^]^. Ozaki et al.^[^4^]^ have also reported favorable outcomes of AVNeo surgery in patients with BAV and no adverse events in seven patients with IE without PA, who were < 60 years old after a mean follow-up of 34 months^[^5^]^.

Currently, the ideal treatment for young IE patients is a topic still debated. The American Association for Thoracic Surgery guidelines advise homograft implantation in cases like this described (Class IIa, level of evidence B)^[^6^]^. Nevertheless, we did not implant a homograft due to unviability.

Therefore, we had to decide between prosthetic valve replacement or AVNeo. In the present case, young age of the patient and specific contraindication to chronic oral anticoagulation due to social issues were considered contraindication to AV replacement with either a biological or a mechanical prosthesis. Furthermore, the durability of most of biological valves in young patients is comparable to the mid-term result of Ozaki follow-up^[^7^]^. At the end, we were also worried about the possible risk of reinfection due to the large PA and the presence of a biological prosthesis’ sewing cuff, which might render a reoperation particularly challenging.

AVNeo surgery appeared particularly appealing in our patient since any prosthetic material was avoided. In fact, autologous pericardium may be less prone to infection, may make antibiotic treatment more effective, preserve the hemodynamic of the aortic root, and, hopefully, will provide an adequate durability.

AVNeo expert opinions retain that the goal of the procedure is a symmetric tricuspidization. The procedure may be also considered in IE, but not in annular abscess; in the latter case, however, there is no evidence.

Nevertheless, we obtained an asymmetric valve due to the more complex valve anatomy of BAV Sievers type 0 A-P with the dislocation of the coronary ostia, which render tricuspidization more difficult.

Therefore, our case report demonstrates that AVNeo with autologous pericardium in young patients with AV IE complicated with PA may be considered a valid alternative to biological prosthesis due to its clinical advantages considering short-term follow-up.
